# Opposite functions of RapA and RapC in cell adhesion and migration in *Dictyostelium*

**DOI:** 10.1080/19768354.2021.1947372

**Published:** 2021-07-01

**Authors:** Jihyeon Jeon, Dongju Kim, Taeck Joong Jeon

**Affiliations:** Department of Biology & BK21- Plus Research Team for Bioactive Control Technology, College of Natural Sciences, Chosun University, Gwangju, Republic of Korea

**Keywords:** RapA, RapC, *Dictyostelium*, Ras proteins, cell adhesion

## Abstract

There are three Rap proteins in *Dictyostelium*. RapA is a key regulator of cell adhesion and cytoskeletal rearrangement. Recently, RapC has been reported to be involved in cytokinesis, cell migration, and multicellular development. Here, we compare the functions of RapA and RapC using cells expressing or lacking Rap proteins, and confirm that RapA and RapC have opposite functions in cell spreading, adhesion, and migration. On the other hand, RapC has a unique function in cytokinesis and multicellular development. Activated RapA appears to stimulate spreading and adhesion of the cells to the substrate, possibly resulting in a decrease in the migration speed of the cells during chemotaxis without affecting the directionality, whereas RapC suppresses cell spreading and adhesion, thereby increasing the migration speed. Cells lacking RapC were defective in cytokinesis and multicellular development and showed multinucleation and formation of multiple tips from a mound during development. At the C-terminus, RapC has an additional stretch of amino acids, which is not found in RapA. The mechanism through which RapA and RapC perform their opposite functions in diverse cellular processes should be characterized further to understand the Rap signaling pathways in detail.

**ABBREVIATIONS:** GAP; GTPase-activating proteins; GEF; guanine nucleotide exchanging factor; WT; wild type; CA; constitutively active; DN; dominantly negative

## Introduction

Ras family proteins play important roles in diverse cellular signaling processes responsible for cell morphology, motility, cell survival, cell cycle progression, and endocytosis (Wennerberg et al. 2005; Raaijmakers and Bos 2009; Simanshu et al. 2017; Wang et al. 2020). The Ras proteins are small GTPases cycling between GTP-bound activated and GDP-bound inactivated forms by GEFs (Guanine nucleotide exchanging factors) and GAPs (GTPase-activating proteins), respectively (Raaijmakers and Bos 2009). In mammalian cells, the Ras family is composed of three Ras (H-Ras, K-Ras, and N-Ras), five Rap (Rap1A, Rap1B, Rap2A, Rap2B, and Rap2C), R-Ras, Ral, and Rheb proteins (Wennerberg et al. 2005; Raaijmakers and Bos 2009). *Dictyostelium discoideum*, a free-living social amoeba, has been used as a model organism to study basic cellular processes including cell migration, phagocytosis, and cytokinesis, as well as the Ras signaling pathways (Kortholt and van Haastert 2008; Hilbi and Kortholt 2017). Most of the signaling molecules involved in the cellular processes are well conserved in *Dictyostelium*. The Ras subfamily consists of 15 proteins in *Dictyostelium*, including eleven Ras (RasB, RasC, RasD, RasG, RasS, RasU, RasV, RasW, RasX, RasY, and RasZ), three Rap (RapA, RapB, and RapC), and one Rheb protein (Kortholt and van Haastert 2008). These Ras proteins are associated with cell motility, cytokinesis, and multicellular development of *Dictyostelium* (Kortholt and van Haastert 2008; Hilbi and Kortholt 2017).

The Rap (RAs-Proximate) proteins were originally identified as Ras antagonists and consist of several isoforms (Kortholt and van Haastert 2008; Raaijmakers and Bos 2009). There are three Rap proteins in *Dictyostelium*. RapA and RapC are close homologs with 51% amino acid sequence identity (Park et al. 2018). RapA is a key regulator of cell adhesion and cytoskeletal rearrangement. Upon chemoattractant stimulation, RapA is transiently and rapidly activated and localizes to the leading edge of migrating cells. Activated RapA causes cell spreading and adhesion by controlling actin cytoskeletal rearrangement and myosin disassembly through the Ser/Thr kinase Phg2 (Jeon et al. 2007b; Lee and Jeon 2012). RapC was recently shown to be involved in cytokinesis, cell migration, and multicellular development in *Dictyostelium*. Loss of RapC resulted in a well-spread morphology with strongly adhesive, multinucleated, and abnormal formation of multicellular structures during development (Park et al. 2018). Intriguingly, RapA and RapC seem to play opposite roles in several cellular processes, such as cell morphology, adhesion, and migration. To investigate the antagonistic functions of RapA and RapC in cellular processes, we prepared cells expressing mutated Rap proteins, including constitutively active (CA) and dominantly negative (DN) forms of RapA and RapC, and compared the phenotypes of these cells to those of wild-type cells and cells lacking RapC (*rapC* null). The results demonstrated that RapA and RapC have opposite effects on cell spreading, adhesion, and migration.

## Materials and methods

### Strains and plasmids

*Dictyostelium* wild-type KAx-3 cells were grown in HL5 medium or in association with *Klebsiella aerogenes* at 22°C. Knock-out strains and transformants were maintained in 10 μg/mL blasticidin and 10 μg/mL G418. The full coding sequences of the *rapA* and *rapC* cDNA were generated by RT–PCR and were cloned into the *Eco*RI – *Xho*I site of the expression vector pEXP-4(+) containing a GFP fragment at the N-terminus (Jeon et al. 2007b; Park et al. 2018). The RapA mutants RapA^CA^ and RapA^DN^ were described previously (Jeon et al. 2007b). A glycine at amino acid 14 was replaced with a valine in RapA^CA^ and a serine at amino acid 19 was replaced with an aspartate in RapA^DN^. The RapC mutants RapC^CA^ and RapC^DN^ were generated using the QuikChange Site-Directed Mutagenesis kit (Stratagene, CA, USA). RapC^CA^ contains a valine at amino acid 13 instead of a glycine in wild-type RapC and RapC^DN^ has an aspartate at amino acid 18 instead of a serine in wild-type RapC. The *rapC* knockout construct and *rapC* null cells were described previously (Park et al. 2018).

### Cell adhesion and chemotaxis analysis

Cell adhesion assay was performed as described previously (Kim et al. 2017). Exponentially growing cells on the plates were harvested and washed with 12 mM Na/K phosphate buffer (pH 6.1), and then 2 × 10^6^ cells were placed on a 6-well culture plate followed by shaking the plates at 150 rpm for 1 h. Total cells and the attached cells after agitating and aspiration of the detached cells were counted. Cell adhesion was presented as a percentage of attached cells compared with total cells.

Chemotaxis toward cAMP was performed as described previously (Park et al. 2018). Aggregation-competent cells were prepared by incubating the cells at a density of 5 × 10^6^ cells/mL in Na/K phosphate buffer for 10 h, and cell migration toward cAMP chemoattractant in a Dunn chemotaxis chamber (Hawksley, Sussex, UK) was recorded at intervals of 1 min for 30 min using an inverted microscope (IX71; Olympus, Tokyo, Japan) with a camera (DS-Fil; Nikon, Tokyo, Japan). The data were analyzed using NIS-Elements software (Nikon) and ImageJ software (National Institutes of Health, Bethesda, MD, USA). The migration speed was calculated by dividing the total distance traveled of a cell with time. Directionality was calculated as the shortest linear distance between the start and end points of the migration path divided by the total distance traveled by a cell for 10 min.

### Nuclei staining and development

Log-phase growing cells were placed on the coverslip and fixed with 3.7% formaldehyde for 10 min. The fixed cells were permeabilized with 0.1% Triton X-100 following staining with 0.2 ug/mL of Hoechst Dye (Sigma) for 20 min (Mun et al. 2014).

Development was performed as described previously (Park et al. 2018). Exponentially growing cells were harvested and washed twice with Na/K phosphate buffer, and then plated on Na/K phosphate agar plates at a density of 3.5 × 10^7^ cells/mL. The images of the muticellular structures were examined and captured with a phase-contrast microscope.

### Statistical analysis

All data were collected from at least three independent experiments and expressed as the means ± standard deviation (SD). Statistical analysis was performed using Student’s *t*-test (two-tailed). *P* values less than 0.05 was considered as statistically significant.

## Results

### Opposite functions of RapA and RapC in cell morphology and adhesion

**Figure 1.**
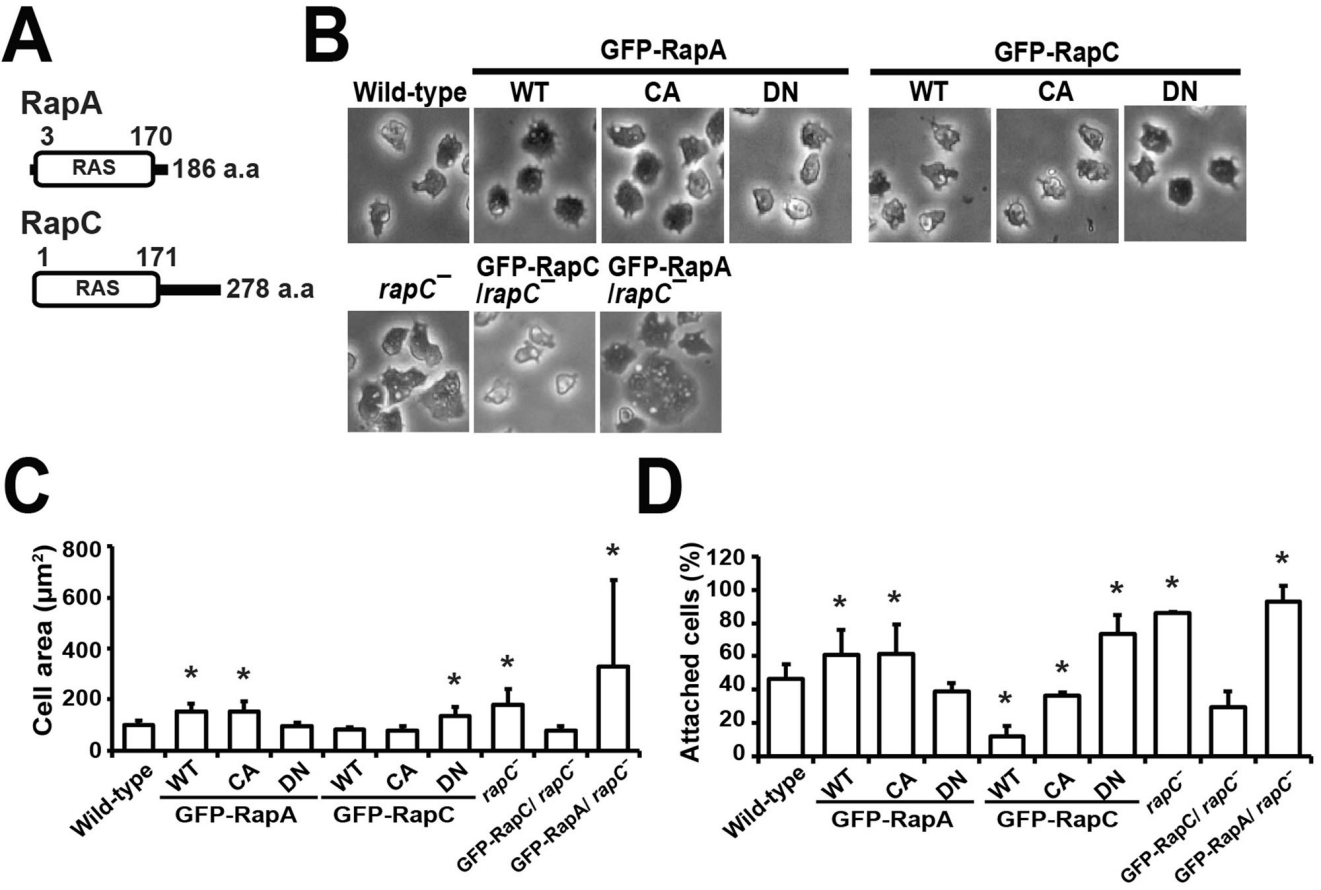
Cell spreading and adhesion. (A) Domain structures of RapA and RapC. (B) Morphology of the cells. GFP-fused mutant proteins for RapA and RapC, constitutively active (CA) and dominantly negative (DN) forms of RapA and RapC, were generated and introduced into wild-type cells. The morphologies of the cells expressing GFP-RapA^WT^, RapA^CA^, RapA^DN^, RapC^WT^, RapC^CA^, and RapC^DN^ were photographed and compared to those of wild-type cells (Upper panel). To determine if the phenotypes of *rapC* null cells are complemented by expression of RapA and RapC, *rapC* null cells expressing wild-type forms of RapA and RapC were prepared and compared to wild-type cells and *rapC* null cells (Lower panel). (C) Quantification of the cell area. The area of the cells were measued using ImageJ software and graphed. The values are the means ± SD of at least three independent experiments. (D) Cell-substrate adhesion. Adhesion of the cells was expressed as a percentage of attached cells to total cells. Error bars represent SD. Statistically different from contol (wild-type) at *p < 0.05 by the student’s *t*-test.

The *Dictyostelium* Ras subfamily comprises 15 proteins including three Rap proteins, RapA, RapB, and RapC (Kortholt and van Haastert 2008; Park et al. 2018). RapA and RapC are composed of 186 and 278 amino acids, respectively, and share 51% identity in the amino acids that constitute the Ras domain. RapC has a stretch of additional amino acid residues at the C-terminus (Figure 1(A)).

To compare the roles of RapA and RapC in cellular processes, we constructed CA and DN forms of RapA and RapC, and cells expressing these GFP-fused mutant proteins as well as cells expressing the wild-type (WT) form of RapA and RapC. Cells expressing RapA^WT^ or RapA^CA^ showed flat and well-spread morphology and approximately 1.5-fold increase in cell size compared to wild-type cells, whereas RapA^DN^ cells displayed decreased cell size (Figure 1(B, C)). In contrast, *rapC* null and RapC^DN^ cells were similar to RapA^WT^ and RapA^CA^ cells both morphologically and in terms of cell size. RapC^WT^ and RapC^CA^ cells exhibited decreased cell size, like the RapA^DN^ cells (Figure 1(B, C)). These results indicate that RapA drives cell spreading and increase in cell size, whereas RapC causes the cells to shrink and decrease in size.

Next, to examine the roles of RapA and RapC in cell adhesion, we measured the percentage of attached cells per total number of cells in the plate after agitating the plates for 1 h followed by aspiration of the detached cells (Figure 1(D)). Similar to the results of cell spreading, RapA^WT^, RapA^CA^, RapC^DN^, and *rapC* null cells showed increased cell adhesion, whereas RapA^DN^, RapC^WT^, and RapC^CA^ cells displayed decreased cell adhesion, compared to wild-type cells. These results suggest that RapA and RapC have opposite roles in cell adhesion; RapA increases the strength of cell adhesion, whereas RapC decreases it.

To further confirm the opposite functions of RapA and RapC in cell spreading and adhesion, we sought to determine whether the phenotype of *rapC* null cells was rescued by expression of RapA and RapC (Figure 1). *rapC* null cells were spread and adhesive to the substrate compared to wild-type cells. This phenotype of *rapC* null cells was complemented by expression of RapC but not RapA. The spread shape of *rapC* null cells was restored to the appearance of wild-type cells following expression of GFP-RapC, and *rapC* null cells expressing RapC showed approximately 1.5-fold weaker adhesion compared to the wild-type cells. In contrast, *rapC* null cells expressing RapA exhibited a highly spread morphology and slightly increased cell adhesion compared to *rapC* null cells (Figure 1(D)). These results confirm that RapA has a positive effect, whereas RapC has a negative effect, on cell spreading and adhesion.

### Opposite roles of RapA and RapC in cell migration

**Figure 2.**
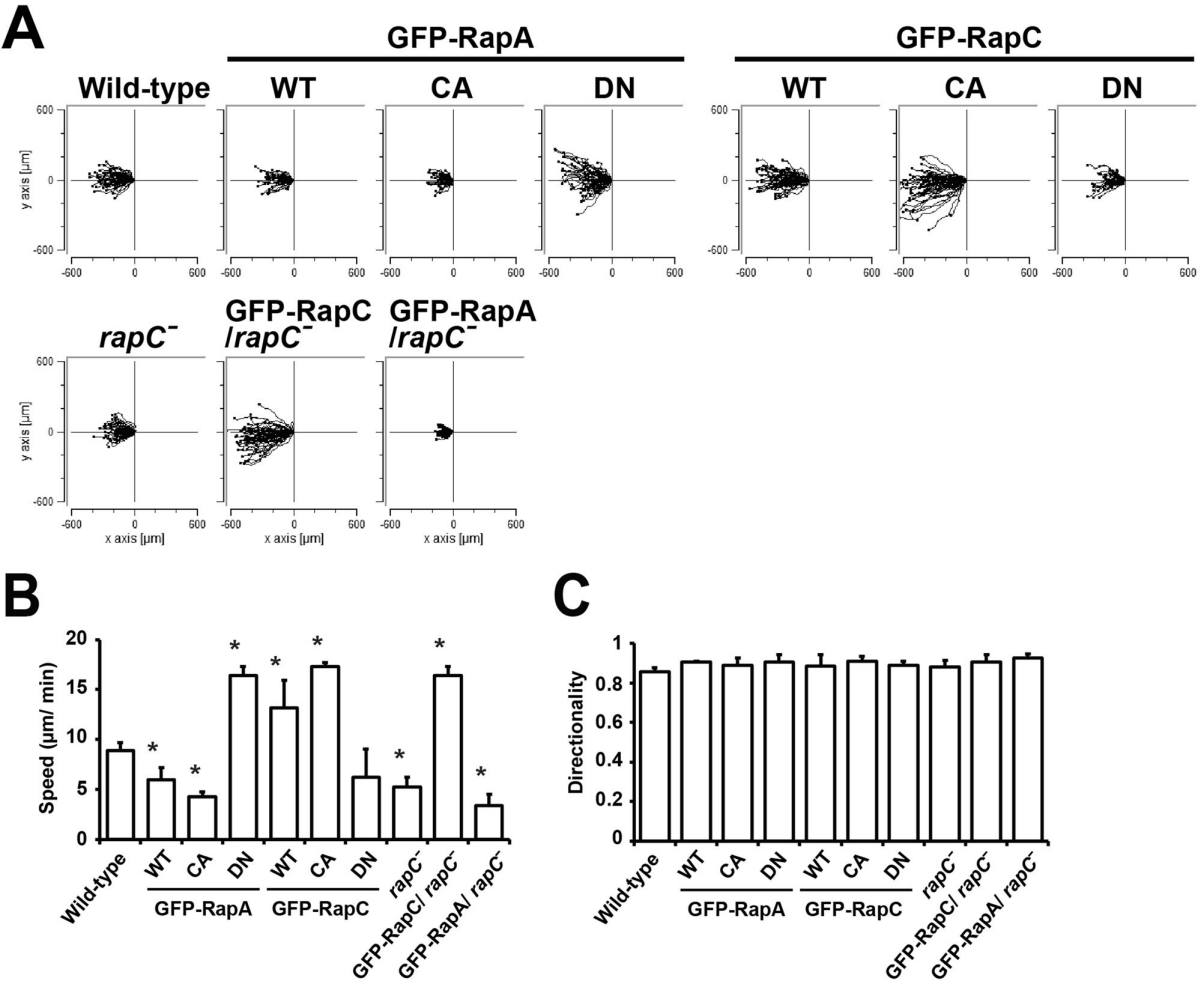
Chemotaxis. (A) Trajectories of cells moving toward cAMP chemoattractants. Aggregation competent cells were placed in a gradient of cAMP in a Dunn chemotaxis chamber, and movements of the cells were recorded at intervals of 1 min for 30 min. Migration paths of the cells were tracked using ImageJ software. Plots show migration paths of the cells with the start position of each cell centered at point 0.0. (B) Quantification of cell motility. Trajectory speed indicates the speed of the cell’s movement along the total path. (C) Directionality is a measure of how straight the cells move. Cells moving in a straight line have a directionality of 1.0. Error bars represent SD from three independent experiments. Statistically different from contol (wild-type) at *p < 0.05 by the student’s *t*-test.

Cell migration requires coordinated and dynamic regulation of cell adhesion. Our previous results indicate that cell adhesion is regulated by RapA and RapC in opposite ways. To determine the roles of RapA and RapC in cell migration, we examined the chemotactic ability of cells expressing RapA or RapC toward cAMP chemoattractants using a Dunn chemotaxis chamber (Figure 2). Consistent with the results of cell adhesion assays, all cells with strong adhesive properties, including RapA^WT^, RapA^CA^, RapC^DN^, and *rapC* null cells, moved slower compared to wild-type cells, while the cells showing weak adhesion, including RapA^DN^, RapC^WT^, and RapC^CA^ cells, exhibited increased speed of migration (Figure 2(B)). There was no significant difference in the directionality of the cells moving toward high concentrations of cAMP (Figure 2(C)). These results indicate that RapA and RapC have opposite functions in cell migration. RapA appears to decrease the migration speed during chemotaxis toward the chemoattractant cAMP, possibly by increasing cell adhesion to the plates. In contrast, RapC appears to increase the migration speed, possibly by decreasing cell adhesion. The effect of cell adhesion on the control of migration speed should be further characterized.

*rapC* null cells showed strong adhesion and decreased migration speed compared to wild-type cells. To confirm the opposite functions of RapA and RapC in cell migration, we examined whether the phenotype of *rapC* null cells during chemotaxis was complemented by expression of RapA and RapC (Figure 2). When RapC was introduced into *rapC* null cells, the migration speed increased more than that of wild-type cells, whereas *rapC* null cells expressing RapA showed markedly decreased migration speed (Figure 2(B)). In terms of directionality, there was no significant difference among the cells (Figure 2(C)). These results support the previous observation that; RapA decreases the migration speed during chemotaxis, whereas RapC increases it.

### Rapc is involved in cytokinesis

**Figure 3.**
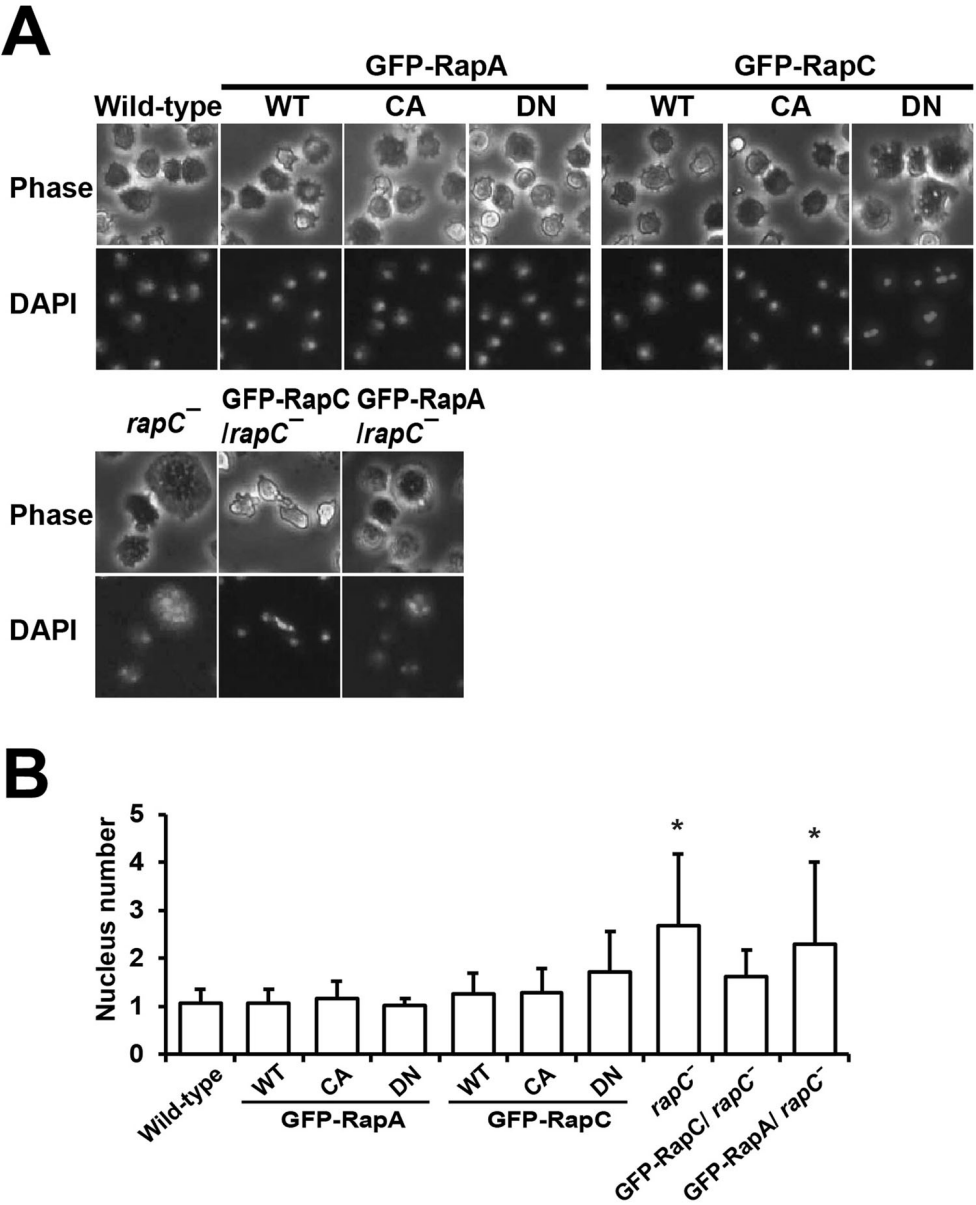
Cytokinesis. (A) Representative phase-contrast and DAPI images of the cells. Exponentially growing cells were stained with a Hoechst dye and photographed. (B) Quantification of the number of nucleus in the cells. Error bars represent SD from three independent experiments and *n* = 100 cells for each cell line. Statistically different from contol (wild-type) at *p < 0.05 by the student’s *t*-test.

Loss of RapC causes defects in cytokinesis and *rapC* null cells are multinucleated (Park et al. 2018). To compare the functions of RapA and RapC in cytokinesis, cells expressing RapA- or RapC-mutant proteins were stained with a Hoechst dye, and the nuclei were counted (Figure 3). All cells except *rapC* null cells and cells expressing RapC^DN^ contained approximately one nucleus per cell. In contrast, *rapC* null cells contained 2–3 nuclei per cell, and cells expressing RapC^DN^ contained approximately two nuclei per cell (Figure 3(A, B)). The multinucleated phenotypes of *rapC* null cells were rescued by expressing RapC but not RapA (Figure 3). *rapC* null cells expressing RapC had approximately one nucleus per cell similar to the wild-type cells. These results suggest that RapC, but not RapA, plays a specific role in cytokinesis.

### RapC is required for proper multicellular development

**Figure 4.**
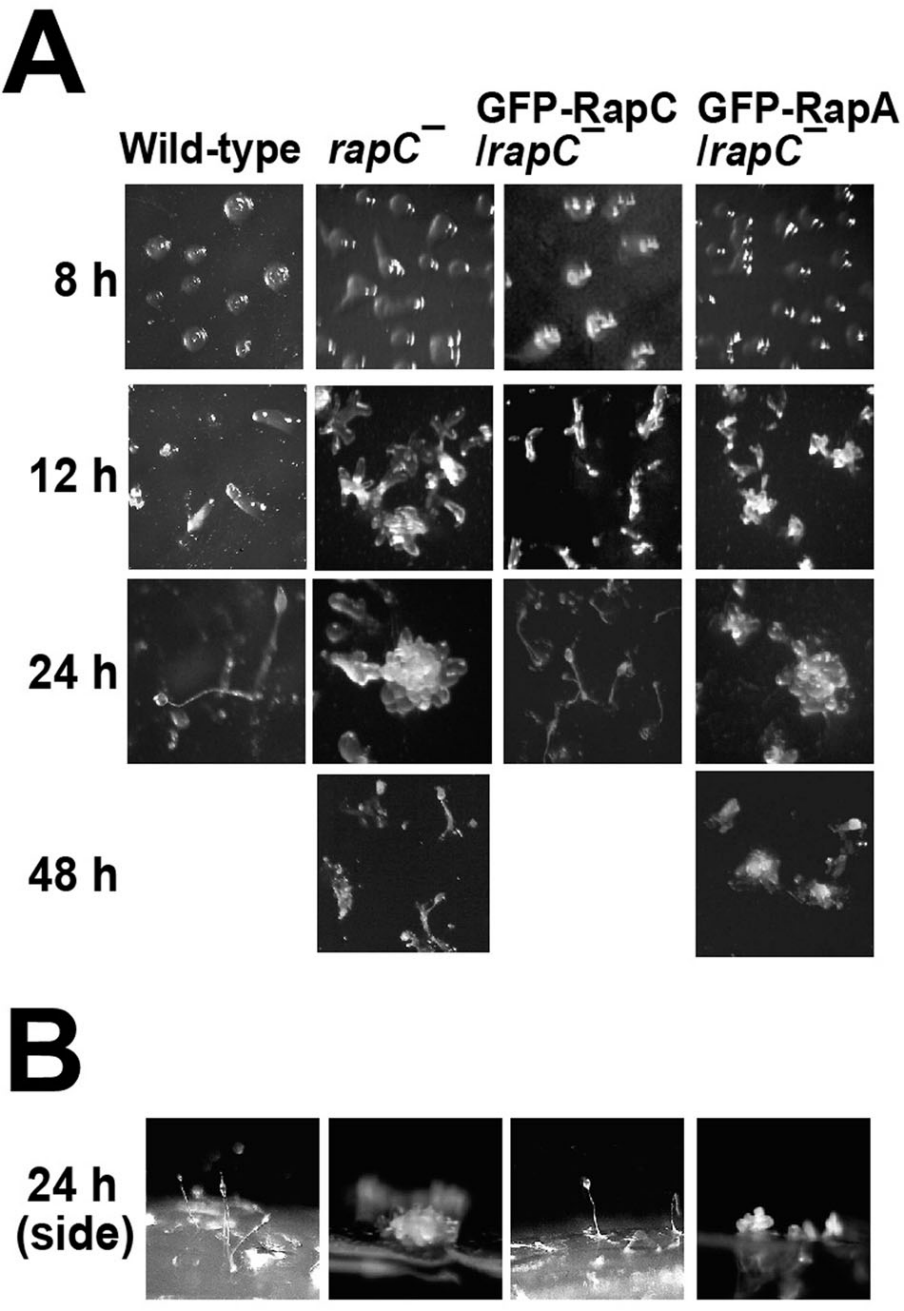
Multicellular development. (A) Developmental morphology of the cells. Exponentially growing cells were developed on non-nutrient agar plates and representative developmental images at the developmental stages were presented. (B) Side view of multicellular structures at 24 h development.

When *Dictyostelium* cells are starved, the cells aggregate and differentiate into several types of cells. Then they finally form fruiting bodies consisting of a dead stalk with a mass of spores on the top (Chisholm and Firtel 2004). To investigate the possible roles of RapA and RapC during multicelluar development, we compared the multicellular structures that were formed by cells expressing RapA or RapC during *Dictyostelium* development (Figure 4). Wild-type cells formed aggregates at 8 h after induction of development, slugs at approximately 12 h, and fruiting bodies within 24 h. All cells expressing RapA, RapC, or mutated Rap proteins proceeded to develop normally although there was a slight difference in the timing of the formation of each structure (Data not shown). However, unique developmental defects were found in *rapC* null cells (Figure 4). Cells lacking RapC formed multiple tips on mounds and multiple developmental structures from a single mound, contrary to formation of one tip from one mound in the wild-type cells. This phenotype of *rapC* null cells was rescued by the expression of RapC, but not RapA (Figure 4). These results indicate that RapC has a specific role in the control of tip formation from a mound during multicellular development in *Dictyostelium*.

## Discussion

This study demonstrates that RapA and RapC have opposite functions in controlling cell morphology, adhesion, and migration in *Dictyostelium*. Activated RapA appears to stimulate spreading and adhesion of the cells to the substrate, decreasing the migration speed during chemotaxis, whereas RapC suppresses cell spreading and adhesion, thus increasing the migration speed. On the other hand, RapC appears to have a unique function in cytokinesis and multicellular development. When the levels of wild-type or CA form of RapA were increased, the cells were flat and adhesive and moved slowly toward the chemoattractant. In contrast, cells containing elevated levels of wild-type or CA form of RapC were shrunk and displayed weak adhesion to the plate and high migration speed compared to the wild-type cells. In agreement with these findings, the phenotypes of *rapC* null cells that were similar to cells with high levels of RapA were restored by RapC but not RapA. *rapC* null cells expressing RapA showed increased spread morphology and stronger adhesion compared to *rapC* null cells or wild-type cells expressing RapA. We were unable to examine the phenotypes of cells lacking RapA since *rapA* null cells are unavailable, suggested that RapA is essential (Jeon et al. 2007b).

RapA is a key regulator of integrin-mediated cell adhesion and cadherin-mediated cell–cell interaction and has been linked to several signaling pathways, including cell migration, osmotic stress responses, and phagocytosis; additionally, it functions as a regulator of cytoskeletal rearrangements during cell migration (Kortholt and van Haastert 2008; Lee and Jeon 2012; Hilbi and Kortholt 2017; Park et al. 2020). RapA is activated in response to chemoattractant stimulation, and activated RapA mediates cell adhesion through Phg2 Ser/Thr kinase and myosin II disassembly at the leading edge of migrating cells (Jeon et al. 2007b). Recently, RapA was shown to directly bind to the RA domain of talin and RacGEFA, thereby inducing F-actin assembly and increasing the strength of cell adhesion (Mun and Jeon 2012; Plak et al. 2016). TORC2 plays an important role in regulating cytoskeleton dynamics and cell migration. Rap1 has been reported to positively regulate the RasC-mediated activation of TORC2 (Khanna et al. 2016). Our data demonstrate that RapA and RapC affect migration speed but not directional sensing during chemotaxis. RapA appears to decrease the migration speed, whereas RapC increases the speed without affecting the directionality of the cells during chemotaxis. Decreased migration speed during chemotaxis was also observed in cells overexpressing a GEF protein specific to RapA (GbpD) and in *rapGAP1* or *rapGAP9* null cells (Kortholt et al. 2006; Jeon et al. 2007a; Mun et al. 2014). These cells have similarly high levels of activated RapA, supporting the notion that proper regulation of the activity of RapA is required for directed cell migration. However, it is unknown whether RapC is activated by chemoattractants and whether the activity of RapC is regulated through GbpD, RapGAP1, and RapGAP9. Further study of the regulation of RapC activity in response to external stimuli, such as chemoattractants, would provide better insights into the antagonistic effects of RapA and RapC on cell spreading, adhesion and migration.

In contrast to RapA, RapC has unique functions in cytokinesis and multicellular development. Cells lacking RapC or expressing a DN form of RapC displayed multinucleation. In addition, *rapC* null cells showed defects in multicellular development, forming multiple tips and structures from a single mound. These phenotypes were restored by RapC but not by RapA. In our study, cells expressing RapA or RapA-mutant proteins demonstrated no defect in cytokinesis and development, although another group reported that cells overexpressing CA form of RapA were defective in cytokinesis and multinucleated (Plak et al. 2014), and some cells showed slightly delayed developmental processes. Similar multinucleation and cytokinesis defects have been reported in cells lacking RapGAP9 (Mun et al. 2014). In contrast, other RapA-related mutants, including cells overexpressing or lacking GbpD and RapGAP1, displayed normal cytokinesis and development (Kortholt et al. 2006; Jeon et al. 2007a). The developmental defect of *rapC* null cells resulting in the formation of multiple structures from a mound are likely to be generated by misregulation of cAMP waves. Tip formation from aggregates during multicellular development is controlled by cAMP waves, propagating from the top to the posterior of the multicelluar aggregates and inducing differentiation and sorting of the cells in the mounds into prespore and several cell types (Siegert and Weijer 1992, 1995). Similar developmental phenotypes were found in the presence of caffeine, an inhibitor of adenylyl cyclase (Siegert and Weijer 1992; Tariqul Islam et al. 2019). RapGAP3, a RapA-specific GAP protein, plays an important role in regulating cell sorting during apical tip formation through controlling the activity of RapA at the late mound stage of development (Jeon et al. 2009). RapC has an opposite function on cell adhesion to RapA. The developmental defect of *rapC* null cells of multiple tip formation might be resulted from disturbed regulation of cell–cell adhesion in the multicellular organisms.

This study confirms that, despite their high sequence homology, RapA and RapC have opposite functions in cell spreading and adhesion. RapC appears to have a unique function in cytokinesis and multicellular development. At the C-terminus, RapC has an additional stretch of amino acids, which is not found in RapA. The mechanism through which RapA and RapC perform their opposite functions in diverse cellular processes needs to be characterized further to understand the Rap signaling pathways in detail.

## Supplementary Material

Supplemental Material
