# Supplementary figures and images for: Opposite functions of RapA and RapC in cell adhesion and migration in Dictyostelium
Source: Anim Cells Syst (Seoul). 2021 Jul 1;25(4):203–10. doi: 10.1080/19768354.2021.1947372 (PMC8370755; doi:10.1080/19768354.2021.1947372)

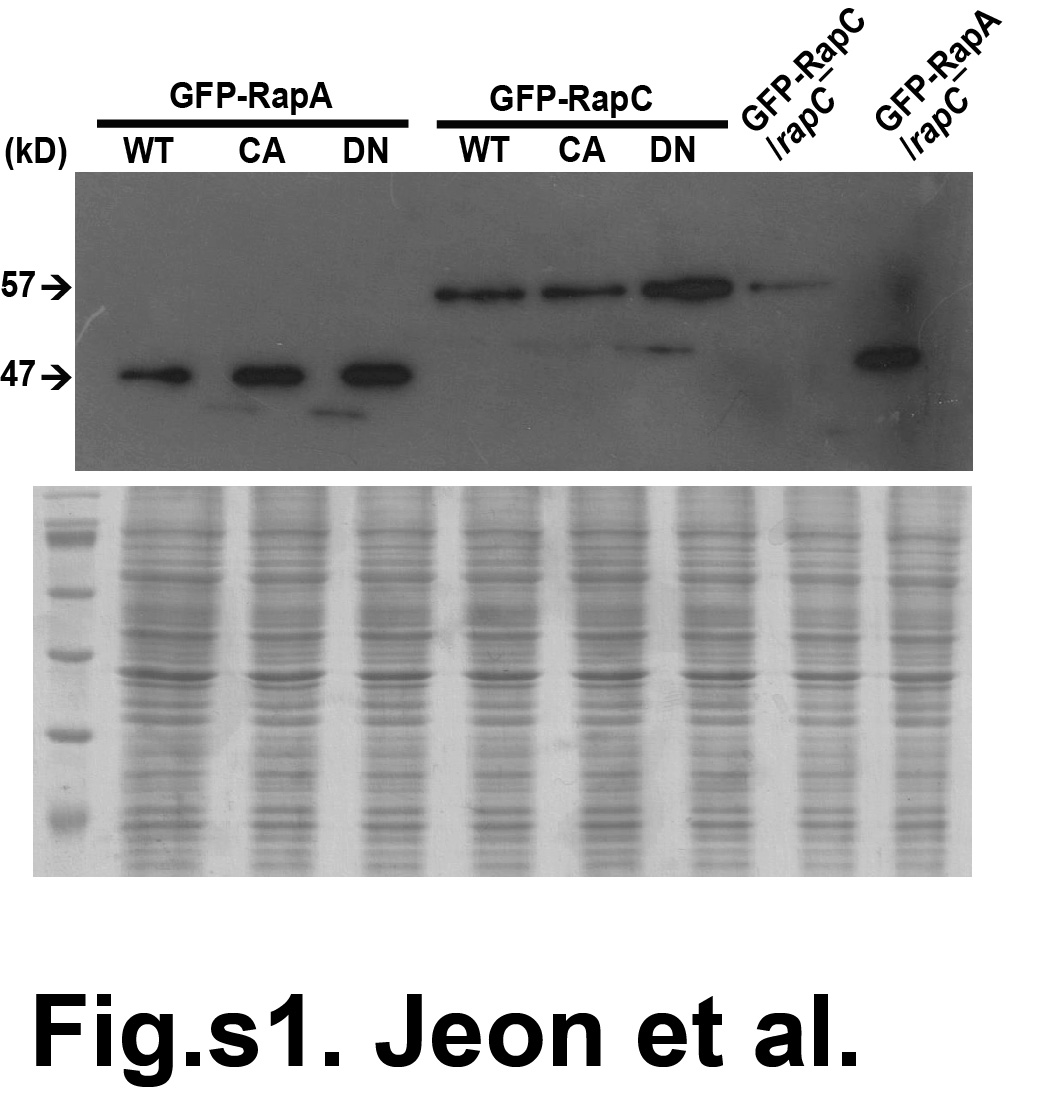

Supplement: Supplemental Material [file TACS_A_1947372_SM8494.jpg]
